# Cell-Free Enzymatic Conversion of Spent Coffee Grounds Into the Platform Chemical Lactic Acid

**DOI:** 10.3389/fbioe.2019.00389

**Published:** 2019-12-03

**Authors:** Dominik Kopp, Robert D. Willows, Anwar Sunna

**Affiliations:** ^1^Department of Molecular Sciences, Macquarie University, Sydney, NSW, Australia; ^2^Biomolecular Discovery and Design Research Centre, Macquarie University, Sydney, NSW, Australia

**Keywords:** enzyme biocatalysis, mannose, cell-free, spent coffee grounds, lactic acid, NMR flux analysis, metabolic engineering, waste

## Abstract

The coffee industry produces over 10 billion kg beans per year and generates high amounts of different waste products. Spent coffee grounds (SCG) are an industrially underutilized waste resource, which is rich in the polysaccharide galactomannan, a polysaccharide consisting of a mannose backbone with galactose side groups. Here, we present a cell-free reaction cascade for the conversion of mannose, the most abundant sugar in SCG, into L-lactic acid. The enzymatic conversion is based on a so far unknown oxidative mannose metabolism from *Thermoplasma acidophilum* and uses a previously characterized mannonate dehydratase to convert mannose into lactic acid via 4 enzymatic reactions. In comparison to known *in vivo* metabolisms the bioconversion is free of phosphorylated intermediates and cofactors. Assessment of enzymes, adjustment of enzyme loadings, substrate and cofactor concentrations, and buffer ionic strength allowed the identification of crucial reaction parameters and bottlenecks. Moreover, reactions with isotope labeled mannose enabled the monitoring of pathway intermediates and revealed a reverse flux in the conversion process. Finally, 4.4 ± 0.1 mM lactic acid was produced from 14.57 ± 0.7 mM SCG-derived mannose. While the conversion efficiency of the process can be further improved by enzyme engineering, the reaction demonstrates the first multi-enzyme cascade for the bioconversion of SCG.

## Introduction

The biotechnological production of versatile chemicals in a sustainable manner depends on the availability of low-cost biomass and efficient microbial production hosts. Advances such as cost-efficient gene synthesis, refactoring metabolic pathways using standardized synthetic building blocks and genome scale metabolic models allow more predictable modifications of cellular hosts. However, key challenges such as internal regulatory mechanisms, metabolic burden on a growing cell and chemical sensitivity of living cells limit the design opportunities for a truly synthetic approach to metabolic engineering. Cell-free biocatalysis has the potential to overcome a variety of challenges encountered during *in vivo* metabolic engineering (Dudley et al., 2015; Morgado et al., 2016; Petroll et al., 2018). In cell-free biocatalysis, purified enzymes or cell lysates are assembled into synthetic multi-enzyme cascade reactions, which can be rapidly tested and easily adjusted. The cell-free design allows for unrestricted combination of different enzymes and a truly synthetic approach to metabolic engineering. Common obstacles of *in vivo* biosynthesis of chemicals like product toxicity, limited cellular uptake, and secretion or genetic compatibility with the production host can be overcome in a cell-free environment. To date, this cell-free approach has been successfully applied to produce a range of different bulk and speciality chemicals including iso- and n-butanol, ethanol, hydrogen, α-ketoglutarate, mevalonate, and dihydroxyacetone phosphate (Ye et al., 2009; Guterl et al., 2012; Krutsakorn et al., 2013; Dudley et al., 2016; Hold et al., 2016; Beer et al., 2017). Besides the immense diversity in enzyme combinations, mostly refined substrates like glucose have been used as starting substrates for these processes. The drive toward a lower ecological footprint, further reduction of costs and avoidance of competition with food resources increased the efforts toward non-edible or wasted biomass. More recently, alternative feedstocks to hydrolysates from lignocellulosic biomass, such as chitin, corn steep water, or glycerol have been implemented in cell-free bioconversions for the production of chemicals and fuels (Gao et al., 2015; Honda et al., 2017; Li et al., 2017).

Coffee is one of the world's largest traded commodities, with an estimated production of over 10 billion kg per year (International Coffee Organization, 2019). Spent coffee grounds (SCG) are the primary by-products of coffee production, which, despite their high-value components, are disposed mostly to landfill. Approaches to reutilize some of this waste have been undertaken to produce drop-in biofuels via oil extraction (Caetano et al., 2012; Vardon et al., 2013), as a source for bioactive compounds and antioxidants (Ramalakshmi et al., 2009), for the extraction of immunostimulatory oligosaccharides (Takao et al., 2006) and the extraction of phenolic compounds (Zuorro and Lavecchia, 2012). Various fungal strains have been cultivated on SCG for the production of ethanol and the extraction of polyphenolic compounds (Machado et al., 2012; Mussatto et al., 2012). Several different microbial species have been employed for the production of polyhydroxyalkanoates and carotenoids from oil and sugars in SCG (Obruca et al., 2015). Recently, acid-hydrolyzed SCG was used to cultivate several strains of lactic acid bacteria (Hudeckova et al., 2018). However, fermentation with microbial strains, can be impaired by toxic compounds such as polyphenols, tannins and caffeine present in SCG and results in limited yields. Although on average 50% of SCG is composed of carbohydrates, only limited research has been undertaken to exploit specifically this component of SCG (Campos-Vega et al., 2015).

**Figure 1:**
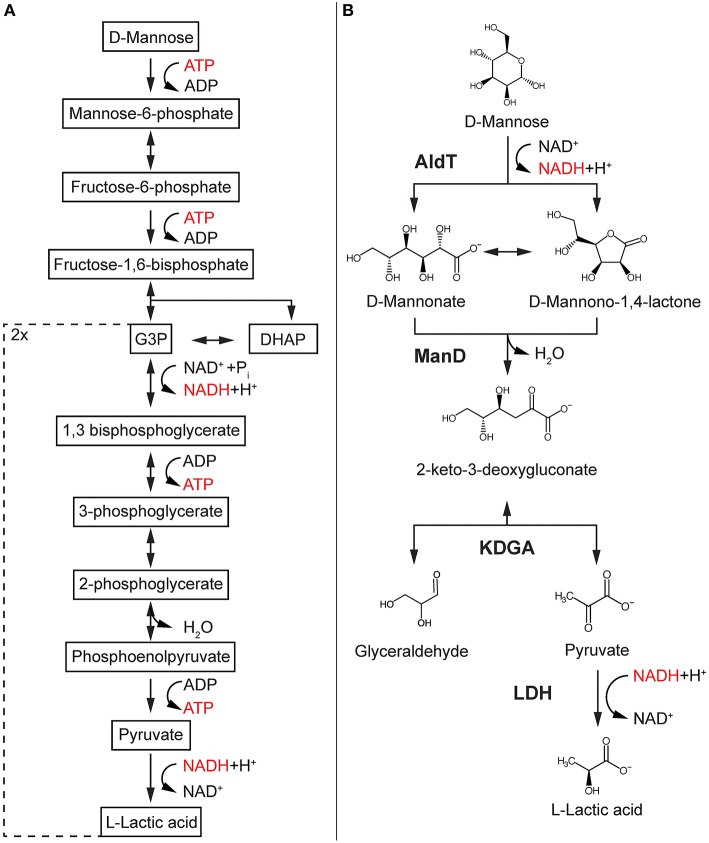
Glycolysis-based mannose pathway and the designed synthetic reaction cascade. EMP-based pathway found in most production hosts such as *S. cerevisiae* **(A)** compared to synthetic reaction cascade for cell-free conversion of mannose into LA **(B)**. Enzymes are in bold, reduced cofactor in red. AldT, aldohexose dehydrogenase; ManD, mannonate dehydratase; KDGA, 2-keto-3-deoxygluconate aldolase; LDH, lactate dehydrogenase; G3P, glyceraldehyde-3-phosphate; DHAP, dihydroacetone phosphate.

Mannose is the most abundant sugar in hydrolyzed SCG and can be used by many organisms as a growth substrate (Mussatto et al., 2011; Machado et al., 2012). Conventionally, mannose catabolism in industrial production strains like *Saccharomyces cerevisiae* follows the classical Embden-Meyerhof-Parnas (EMP) pathway and comprises at least 10 enzymatic reactions, two different cofactors, heat labile triosephosphates and unfavorable isomerization reactions (Figure 1A). Since the EMP pathway marks a central part of carbon metabolism in the cell, it is usually highly regulated and modifications affect central metabolism and cellular viability. Non-phosphorylative or oxidative pathways such as the non-phosphorylative Entner-Doudoroff (np-ED) pathway do not use ATP, rely on a lower enzyme cost and display a more thermodynamically driven design which result in a high flux compared to the EMP pathway (Flamholz et al., 2013). Despite the discovery of oxidative pathways for many sugars, such as glucose, galactose, xylose, rhamnose, and arabinose and their implementation into production hosts, a mannose-specific pathway has not been discovered yet (Bräsen et al., 2014).

Here, we report the design of a functional non-phosphorylative synthetic cascade reaction to convert mannose obtained from SCG into the valuable platform chemical lactic acid (LA) (Figure 1B). LA is a versatile chemical with a wide range of different applications in medical, textile and food industries and has received most of its attention for the production of the biodegradable polymer polylactic acid (PLA) (Dusselier et al., 2013; Castro-Aguirre et al., 2016). Key enzymes of the novel multi-enzyme cascade reaction include a newly identified mannonate dehydratase and a 2-keto-3-deoxygluconate (KDG) aldolase from the thermoacidophilic archaeon *Thermoplasma acidophilum*. The synthetic cascade reaction not only demonstrates an efficient conversion of mannose derived from SCG, but also indicates a possible mannose metabolism in *Thermoplasma acidophilum*.

## Materials and Methods

### Reagents and Chemicals

All chemicals were purchased from Sigma Aldrich (St. Louis, USA) unless otherwise stated. D-[1,6-^13^C_2_]mannose was obtained from Omicron Biochemicals, Inc. (South Bend, USA), D-mannono-1,4-lactone was obtained from TCI Chemicals (Tokyo, Japan) and was either prepared in water or hydrolyzed according to Lamble et al. (2004) to obtain the free sugar acid D-mannonate. Briefly, for hydrolysis, 1 M stock solutions of D-mannono-1,4-lactone were prepared in 1 M NaOH and incubated at room temperature for 1 h before dilutions were prepared in 50 mM 4-(2-hydroxyethyl)-1-piperazineethanesulfonic acid buffer (HEPES) pH 7.0.

### Bacterial Strains and Plasmids

All DNA preparations, manipulations and digestions were carried out according to Russell and Sambrook (2001). DNA fragments encoding the different proteins were amplified by polymerase chain reaction (PCR) using the primer pairs described in Table S1. The *Escherichia coli* strains used in this study were *E. coli* α-select cells (Bioline, Sydney, AUS), *E. coli* BL21 (DE3) cells (NEB, Ipswitch, USA), and *E. coli* BL21-CodonPlus (DE3)-RIL (Agilent Technologies, Santa Clara, USA). The plasmids used were pETDuet-1 (Novagen, Madison, USA) and pProEX HTc (Invitrogen, Carlsbad, USA). All *E. coli* strains were grown at 37°C in lysogeny broth (LB; 10 g/L tryptone, 5 g/L yeast extract, 10 g/L NaCl) supplemented with 100 μg/ml carbenicillin except for strain BL21-CodonPlus (DE3)-RIL which was supplemented with 100 μg/ml carbenicillin and 50 μg/ml chloramphenicol (final concentrations). To generate the expression plasmids pETDuet-1-AldT (encoding the aldohexose dehydrogenase, AldT) and pProEx HTc-Ta1157 (encoding the 2-keto-3-deoxygluconate aldolase, KDGA), the genes Ta0754 and Ta1157 were amplified directly from *Thermoplasma acidophilum* DSM 1728 (DSMZ, Braunschweig, Germany) genomic DNA using the primers in Table S1. The amplified Ta0754 DNA fragment was digested with BamHI/EcoRI and ligated into similarly cut pETDuet-1 vector. The Ta1157 DNA fragment was digested with EcoRI/HindIII and ligated into similarly cut pProEX HTc vector. To generate the expression plasmid pETDuet-1-BsLDH (encoding the lactate dehydrogenase from *Bacillus stearothermophilus*, LDH*)*, the *ldh* gene sequence (KEGG entry: GT50_12230) was synthesized as a gBlock from IDT DNA Technologies (Singapore). The DNA was amplified by PCR, digested with BamHI/HindIII and ligated into similarly cut pETDuet-1 vector. The expression plasmids were used to transform chemically competent *E. coli* α-select (Bioline).

The expression plasmid for ManD, pProEx Hta-Ta0753 was constructed previously and is described elsewhere (Kopp et al., 2019).

### Production and Purification of Recombinant Proteins

Expressions of AldT and LDH were performed in *E. coli* BL21 (DE3) cells, while ManD and KDGA were expressed in BL21-CodonPlus (DE3)-RIL cells. All strains were grown at 37°C to an OD_600_ of 0.4–0.7, before expression was induced by addition of 0.4 mM Isopropyl β-D-1-thiogalactopyranoside (IPTG). All expressions were performed at 20°C for at least 18 h except for the expression of LDH, which was performed at 37°C for 4 h. Cells were harvested by centrifugation at 5,000 × *g* for 20 min at 4°C. Harvested cells were resuspended in 50 mM HEPES buffer (pH 7.0) and ruptured by three passages through a French pressure cell. Soluble fraction of each lysate was obtained by centrifugation at 15,000 × *g* for 20 min at 4°C. The final soluble fractions containing the His-tagged proteins were loaded onto a 5 ml Ni-NTA affinity column (GE Healthcare) previously equilibrated with buffer containing 50 mM sodium phosphate buffer (NaP), 300 mM NaCl, and 20 mM imidazole, pH 8.0. The column was washed extensively with the same buffer containing 40 mM imidazole before enzymes were eluted isocratically with a final concentration of 400 mM imidazole. Fractions containing the desired enzyme were selected and pooled based on the purification chromatogram acquired at 280 nm. The buffer of eluted enzyme solutions was exchanged to 50 mM HEPES pH 7.0 using an Amicon Ultra centrifugal filter units (4- and 30-kDa cutoff, Millipore). Purity and size of the enzymes were analyzed by sodium dodecyl sulfate-polyacrylamide gel electrophoresis (SDS-PAGE) followed by Coomassie staining. Protein concentrations were determined using a Pierce BCA Protein Assay Kit (Thermo Fisher Scientific, Waltham, USA). All purified enzyme preparations were stored at −80°C.

### Enzyme Assays

For all enzyme assays, reactions were performed in triplicates and all data were plotted and analyzed using Prism 6.0 (GraphPad software, La Jolla, CA). The enzymes AldT, KDGA and LDH were measured at 50°C and 20 mM NaP buffer pH 7.0. AldT kinetics were acquired for the substrate D-mannose, KDGA measured in the direction of aldol addition with pyruvate and glyceraldehyde as substrates and LDH for the substrate pyruvate. For all enzyme measurements one unit of enzyme activity was defined as the amount of enzyme necessary to convert 1 μmol substrate per minute.

AldT activity was assayed in reactions (800 μl) containing between 1 mM and 200 mM D-mannose and 5 mM NAD^+^ in 20 mM NaP buffer pH 7.0. Reaction mixtures were preheated at 50°C for 5 min before enzyme was added to start the reaction. AldT activity was determined photometrically by measuring the increase of NADH levels at 340 nm using a Jasco V-760 UV/Vis spectrophotometer equipped with a heating device PAC-743R set to 50°C. NADH was quantified using the extinction coefficient ε_340_ = 6.22 mM^−1^ cm^−^1. ManD kinetics were acquired previously, which is described elsewhere (Kopp et al., 2019). KDGA activity was assayed in the direction of aldol addition. Reactions (100 μl) were performed in 0.2 ml PCR tubes in a water bath at 50°C and contained 0.5–40 mM pyruvate and D-glyceraldehyde in 20 mM NaP buffer pH 7.0. Reactions were incubated for 15 min before 10 μl of 12.5% (w/v) tricholoroacetic acid (TCA) was added to stop the reaction. Formation of KDG was determined by a modified thiobarbituric acid (TBA) assay (Skoza and Mohos, 1976; Buchanan et al., 1999). Fifty microliter of each stopped reaction was oxidized by addition of 125 μl 25 mM periodic acid in 0.25 M H_2_SO_4_ and incubated at room temperature for 20 min. The oxidation was stopped by addition of 250 μl of 2% (w/v) sodium arsenite in 0.5 M HCl followed by addition of 1 ml of 0.3% TBA before samples were boiled for 10 min. The formation of KDG was measured by reading the absorbance at 549 nm in a Spectrostar Nano plate reader (BMG Labtech, Ortenberg, Germany) and quantified using a molar extinction coefficient of 67.8 mM^−1^ cm^−1^. The activity of LDH was assayed in reactions (500 μl) containing pyruvate (0–50 mM) and 0.2 mM NADH in 20 mM NaP buffer pH 7.0. Reaction mixtures were preheated at 50°C for 200 s before reactions were started by the addition of NADH. LDH activity was determined photometrically by measuring the decrease of NADH at 340 nm in the heated spectrophotometer. NADH was quantified using the extinction coefficient ε_340_ = 6.22 mM^−1^ cm^−1^.

### Sequential Reactions

Sequential reactions were performed with a final concentration of 20 mM mannose, 20 mM NAD^+^, 0.1 mM CoSO_4_ in 50 mM NaP pH 7.0. 6 μl of each enzyme were added to reactions sequentially. Reactions were started with the addition of AldT (0.16 U/ml). ManD (0.07 U/ml) was added after 1.5 h, KDGA (0.51 U/ml) after 16.5 h and LDH (5.8 U/ml) and 10 mM NADH were added after 17.5 h. The complete reaction (AldT+ManD+KDGA+LDH) was terminated after 18.5 h. Sixty microliter of each reaction was stopped with 6 μl 12.5% (w/v) TCA and then subjected to HPLC analysis.

### One-Pot Reactions

Reactions with varying enzyme units were performed in 100 μl volume. Each reaction contained all 4 enzymes (AldT, ManD, KDGA, add LDH), 5 mM mannose, 5 mM NAD^+^, and 0.1 mM CoSO_4_ in 100 mM NaP buffer pH 7.0. Reactions with varying mannose and NAD^+^ concentrations contained 0.2 U/ml AldT, 0.1 U/ml ManD, 0.4 U/ml KDGA, 11.6 U/ml LDH, and 0.1 mM CoSO_4_ in 0.1 M NaP buffer pH 7.0. One-pot reactions were incubated at 50°C for 18 h on a Thermomixer C (Eppendorf) without shaking. Stopping one-pot reactions with 12.5% TCA and analysis on an Agilent Hi-Plex H^+^ as performed in residual activity and sequential pathway tests, led to unsatisfactory results for prolonged one-pot reactions. Instead a different method to precipitate proteins was applied that has been successfully used before (Xie et al., 2018). To stop reactions, 35 μl of 1.9 M HCIO_4_ was added to 65 μl of each sample. This was followed by addition of 13 μl 5 M KOH to neutralize the reaction before samples were centrifuged and analyzed using a Bio-Rad Aminex HPX-87H column (section HPLC Analysis). Reactions for the comparison of LA yields from SCG hydrolysate and from pure mannose as a substrate contained 14.57 ± 0.7 mM and 13.9 ± 0.9 mM mannose, respectively, 3.75 mM NAD^+^, 0.1 mM CoSO_4_, and standard enzyme loading in 0.1 M NaP buffer pH 7.0. Reactions were performed in triplicate at 50°C and after 0, 1, 4, 8, 10, and 18 h incubation time, samples were taken for analysis via HPLC.

### Thermostability of Enzymes

One hundred microliter of each enzyme was incubated at 50°C for 20 h in the absence of substrate but at the same loading (U/ml) as used in the one-pot reactions (0.2 U/ml AldT, 0.1 U/ml ManD, 0.4 U/ml KDGA, 11.6 U/ml LDH). Samples were removed at different times and the residual enzyme activity was measured by spectrophotometric, colorimetric or HPLC analysis. All reactions were performed at least in duplicate.

The residual activity of AldT was assayed in reactions (100 μl) containing 10 mM mannose and NAD^+^ in 20 mM NaP buffer pH 7.0. Reactions were performed for 15 and 45 min before they were stopped by the addition of 10 μl of 12.5% TCA. Samples were then subjected to HPLC.

The residual activity of ManD was assayed in 60 μl reactions containing 10 mM hydrolyzed mannonate and 1 mM CoSO_4_ in 20 mM NaP buffer pH 7.0. The reactions were performed for 1 h before they were stopped by addition of 6 μl 12.5% TCA. Fifty microliter of each sample were analyzed by the TBA method as described in section Enzyme Assays.

The residual activity of KDGA was tested in the aldol cleavage direction, by quantification of pyruvate and glyceraldehyde. Reactions (100 μl) contained 5 mM KDG in 20 mM NaP buffer pH 7.0. Reactions were performed for 15 and 45 min before they were stopped by the addition of 10 μl of 12.5% TCA. Pyruvate formation was measured by subjecting 10 μl of each sample to HPLC analysis.

Remaining activity of LDH was assayed in reactions (100 μl) with 5 mM pyruvate and NADH. Reactions were performed for 10 and 30 min and stopped by the addition of 10 μl 12.5% TCA to the reaction. LA formation was measured by subjecting 10 μl of each sample to HPLC analysis.

For determination of half-lives of AldT and ManD, data points were fitted with Prism 6 (GraphPad software) using non-linear regression with a plateau constrain equal to 0 (Figure S5).

### Hydrolysis of Spent Coffee Grounds

SCG was obtained from a university coffee shop and was dried at 60°C for 1 week to a humidity content of 7.9 ± 0.2% before it was stored 4°C. SCG monosaccharides were extracted by dilute acid hydrolysis according to the standard National Renewable Energy Laboratory (NREL) laboratory analytical procedure described by Sluiter et al. (2008). A set of standard reducing sugars (STD) consisting of 60 mg of D-xylose, D-glucose, D-galactose, L-arabinose, and D-mannose was subjected to the same hydrolysis procedure as SCG in order to correct for any losses of monosaccharides during the procedure. In this process, 3 ml of 72% (v/v) H_2_SO_4_ was added to 300 mg SCG and incubated at 50°C for 1 h under constant stirring (200 rpm) in 150 ml pressure tubes. The solution was then diluted to 4% H_2_SO_4_ by addition of ultrapure water and autoclaved for 1 h at 121°C. After autoclaving, the hydrolysate was filtered and 25 ml of the solution was neutralized by addition of CaCO_3_ until pH 6.0 was reached. The neutralized hydrolysate was washed and filtered through a 0.22 μm sterile filter. The monosaccharide concentration of the filtrate was analyzed by HPLC and quantified using a standard curve of mannose in ultrapure water. The amount of mannose present in SCG was quantified using the final mannose concentration in the hydrolysate corrected for losses observed with the STD. For the use of SCG hydrolysate in the cell-free reaction cascade, hydrolysates were concentrated using a rotational vacuum concentrator (Christ RVC 2-25 CD) to the desired concentration for the use in reaction cascade.

### HPLC Analysis

LA yields were determined using an Agilent 1290 HPLC System (Agilent Technologies, Santa Clara, USA) connected to a diode array detector (DAD) (G4212A) and a refractive index detector (RID) G1362A (Agilent Technologies) heated to 55°C. Chromatographic separation of LA was achieved on a Bio-Rad Aminex HPX-87H organic acid column at 65°C with 5 mM H_2_SO_4_ used as a mobile phase. Standards of L-lactic acid treated equal to the reactions were used for quantification (Figure S6A). The flow rate was adjusted to 0.6 ml/min. Ten to twenty microliter sample was injected for each analysis.

Sequential cascade reactions, separation of intermediates, residual KDGA activity, and residual LDH activity were analyzed on the HPLC systems equipped with an Agilent Hi-Plex H^+^ organic acid column connected to the RID. Standard curves for pyruvate and L-lactic acid were used to determine product formation for residual KDGA and LDH activity, respectively. Yield of the sequential cascade reaction was calculated using the same standard curve for L-lactic acid. 10 mM H_2_SO_4_ was used as a mobile phase at a flow rate of 0.6 ml/min. The column temperature was set to 80°C and the RID heated to 55°C.

Analysis of monosaccharides obtained from SCG dilute acid hydrolysis, residual activity of AldT and quantification of remaining mannose in one-pot reactions was achieved on the HPLC system equipped with a Hi-Plex Pb column (Agilent) connected to a RID. Ultrapure water was used as a mobile phase at a flow rate of 0.6 ml/min. The column temperature was set to 85°C and the RID heated to 55°C. Mannose concentration was calculated using a standard curve prepared with D-mannose standards (Figure S6B).

### NMR Analysis

Nuclear magnetic resonance spectroscopy (NMR) measurements of one-pot reactions were carried out at 50°C (323 K) on a 500 MHz Bruker Avance III HD NMR equipped with a BBFO probe. Twenty percentage D_2_O and 0.4% 10 mM 3-(trimethylsilyl)propionic-2,2,3,3-d4 acid (TMSP) chemical shift standard were added to an enzyme mixture containing 0.2 U/ml AldT, 0.1 U/ml ManD, 0.4 U/ml KDGA, 11.6 U/ml LDH, and a substrate solution containing 10 mM D-[1,6-^13^C_2_]mannose, 10 mM NAD^+^ and 100 mM NaP buffer pH 7.5. Time point zero of the time course reaction was acquired with 500 μl of substrate solution in the absence of enzymes. One-pot reactions were started by combining enzyme mixture and substrate solution. Subsequently reactions were transferred to 5 mm NMR tubes, and 1D power-gated ^1^H decoupled ^13^C NMR spectra were acquired with 16 scans at a pulse angle of 90° with a relaxation delay of 3 s between scans.

Standards for mannonate and mannono-1,4-lactone were prepared by dissolving D-mannono-1,4-lactone in 1 M NaOH or 1 M HCl with incubation for 1 h which was followed by dilution in water to 100 mM. Then, 300 μl of each 100 mM solution (equivalent to 5 mg sugar acid) was freeze-dried and resuspended in 500 μl 100 mM NaP buffer pH 7.5. After transfer of each sample to 5 mm NMR tubes, 10% D_2_O and 40 μM TMSP chemical shift standard was added. For standards, 5 mg of D-glyceraldehyde, pyruvate and L-lactic acid and 1 mg of KDG were each resuspended in 100 mM NaP buffer pH 7.5 to obtain 1D decoupled ^13^C NMR spectra of non-isotopically labeled samples for comparison to reaction intermediates. Spectra of intermediates were acquired using power-gated proton decoupling at a 90° pulse with 512 scans and a 3 s relaxation delay between scans. Since the chemical shift of L-lactic acid changed with pH, the standard was buffered at pH 7.5.

In the labeled time course reaction, C6 compounds (mannose, mannono-1,4-lactone, mannonate and KDG) contained two labeled carbons (carbon 1 and carbon 6). Due to a combination of proton nuclear Overhauser effect (NOE) and shorter relaxation times the carbons with lower chemical shift have higher signal intensities in the decoupled ^13^C NMR. Accordingly, only lower chemical shifts were used for integration (Table S2). Visualization of all spectra was performed with iNMR 6.0. Fourier transformation with an exponential weighting factor of 1.5 was performed followed by automatic phase correction and a smoothing factor of 10 was applied to all spectra. Integrals of chemical shifts changing over time were obtained. Fitting of selected integrals for mannose (61.46 ppm), mannonate (63.52 ppm), mannono-1,4-lactone (63.02 ppm), KDG (64.74 ppm), glyceraldehyde (62.45 ppm), oxidized glyceraldehyde (63.35 ppm), and LA (182.37 ppm) were performed using DynaFit 4.0 (Biokin Ltd., Watertown, USA).

## Results and Discussion

### Design of the Synthetic Reaction Cascade

We designed a cell-free reaction cascade for the bioconversion of LA from mannose (Figure 1B). The first 3 reactions of the cascade are catalyzed by enzymes from the archaeon *T. acidophilum*. In the first reaction step of the reaction cascade, mannose is oxidized via a previously described aldohexose dehydrogenase (AldT) to mannonolactone using NAD^+^ (Nishiya et al., 2014). Mannonolactone hydrolyses spontaneously at neutral pH to mannonate, which is then converted by a mannonate dehydratase (ManD) to 2-keto-3-deoxygluconate (KDG) (Kopp et al., 2019). A KDG aldolase (KDGA) subsequently cleaves KDG into pyruvate and D-glyceraldehyde. Pyruvate is then reduced by a L-lactate dehydrogenase (LDH) from *B. stearothermophilus* with the use of NADH produced in the first oxidation reaction of the reaction cascade. The turnover of NADH is balanced since one mole NADH is produced per mole mannose being oxidized, and one mole NADH is oxidized per mole pyruvate being reduced in the last reaction. Since KDGA converts KDG to equal ratios (50% each) of pyruvate and D-glyceraldehyde, and only pyruvate is converted to LA, the current design of the reaction cascade only allows for 50% of the carbon in the mannose to be converted into LA.

The enzymes used in this reaction cascade were selected according to their temperature and pH compatibility. AldT has been described previously to be stable above pH 7.0 with an optimum activity at pH 10, while retaining full activity after at least 15 min incubation at 60°C (Nishiya et al., 2014). Recently, a putative mannonate dehydratase from *T. acidophilum* (ManD) was recombinantly expressed in *E. coli*, purified and fully characterized (Kopp et al., 2019). ManD shows specific activity toward mannonate and mannono-1,4-lactone at pH 7.0 with a strong decrease in activity above neutral pH. The enzyme showed maximal activity at 65°C and remained stable for at least 1 h at 55°C.

A KDG aldolase in *T. acidophilum* has not been identified before, but indication for the corresponding genes were given based on homology to a KDG aldolase from *Picrophilus torridus* (Reher et al., 2010). Accordingly, the gene locus Ta1157 was recombinantly-produced in *E. coli* and the purified enzyme was shown to successfully cleave KDG to pyruvate and glyceraldehyde. Since this enzyme is also derived from the same organism as AldT and ManD, we assumed a similar temperature optimum and pH range for activity. In the final step of the reaction cascade, the NADH-dependent L-lactate dehydrogenase (LDH) from *B. stearothermophilus* was chosen because of its thermostability (fully active after 20 min at 70°C) at a neutral pH (Murphey et al., 1967).

All single enzyme experiments and the final reaction cascade assemblies were performed with purified enzymes in order to exclude potential side reactions (e.g., cofactor consuming reactions) present in *E. coli* expression lysates (Figure S1).

### Single Enzymes and Reaction Conditions

The kinetic data available for AldT and LDH were acquired previously at 25°C and in different buffer systems (Halliwell et al., 2001; Nishiya et al., 2014). However, the cell-free reaction cascade presented here is intended to function at 50°C. We chose NaP buffer at neutral pH during the selected operating temperature because it faces low temperature dependency and does not interfere with ^13^C NMR. Accordingly, in order to normalize the kinetic data for each enzyme of the reaction cascade, all kinetic assays were performed at 50°C in the same buffer system and a pH of 7.0 (Table 1, Figure S2). Under these conditions, the V_max_ of AldT was only 5.2 ± 0.1 U/mg, which is 4.6-fold lower than that previously reported for AldT (Nishiya et al., 2014). We determined the *K*_m_ value of 9.73 ± 0.78 mM for D-mannose with NAD^+^, which is lower than the *K*_m_ measured previously (D-mannose with NAD^+^ of 25.0 ± 1.8 mM) (Nishiya et al., 2014). Since in both cases enzymes were heterologously-expressed and His-tag purified, the difference is attributed to a different pH and temperature of the assays. ManD was investigated as part of a separate study toward two different substrates, since the oxidation product of mannose is present in an equilibrium of free sugar acid (D-mannonate) and its lactone (D-mannono-1,4-lactone) (Kopp et al., 2019). In general, the enzyme displayed low V_max_ values for both substrates and was the weakest performing enzyme in the process. KDGA from *T. acidophilum* was analyzed in the direction of aldol addition with pyruvate and glyceraldehyde as substrates. The enzyme exhibited a high V_max_ of 17.04 ± 1.03 U/mg which is in a similar range as compared to previously characterized KDG aldolases from different *Sulfolobus* spp. (V_max_ of *S. solfataricus* for pyruvate: 15.7 ± 0.3 U/mg) (Buchanan et al., 1999). The *K*_m_ of the KDGA from *T. acidophilum* for both substrates combined (8.07 ± 1.29 mM) was high but in a comparable range to values reported for the KDG aldolase from *S. solfataricus* (for D,L-glyceraldehyde: 5.2 ± 0.1 mM) (Buchanan et al., 1999). LDH displayed very high specific activity (194.3 ± 11.8 U/mg) for pyruvate that was comparable to those reported previously (241 ± 30 U/mg, Halliwell et al., 2001). However, the *K*_m_ of 10.95 ± 1.67 mM for pyruvate was found to be substantially different from that previously reported (1.65 ± 0.12 mM, Halliwell et al., 2001).

**Table 1:** *K*_m_ and V_max_ values of enzymes used in the cell-free reaction cascade.

**Enzyme**	**Substrate**	***K*_**m**_ [mM]**	**V_**max**_ [U/mg]**	***k*_**cat**_ [s^**−1**^]**	***k*_**cat**_/*K*_**m**_ [mM^**−1**^ s^**−1**^]**	**pH opt**
AldT	Mannose	9.73 ± 0.78	5.20 ± 0.10	2.51	0.26	10^a^
ManD	Mannonate	5.37 ± 0.90	2.39 ± 0.11	1.67	0.30	7.0^b^
	Mannono-1,4-lactone	4.90 ± 0.53	1.90 ± 0.06	1.33	0.26	
KDGA	Pyruvate + glyceraldehyde	8.07 ± 1.29	17.04 ± 1.03	10.51	1.30	n.d.
LDH	Pyruvate	10.95 ± 1.67	194.3 ± 11.8	116.58	10.65	5–7^c^

*Values for AldT, KDGA, LDH were obtained in 20 mM NaP buffer pH 7.0 at 50°C. Kinetic data for ManD was previously obtained at 50°C in 50 mM HEPES pH 7.0 (Kopp et al., 2019). n.d., not determined. Values are means of triplicate experiments and standard deviation (±) is indicated*.

a *Nishiya et al. (2014)*.

b *Kopp et al. (2019)*.

c *Clarke et al. (1988)*.

### Sequential Reactions and Enzyme Validation

Proof-of-concept cascade operation was conducted initially in sequential reactions with D-mannose and an equal molar amount of NAD^+^. Each enzyme of the reaction cascade was added sequentially and individual product accumulation was monitored by HPLC (Figure S3). Besides a clearly separated LA peak, an incomplete separation of all other intermediates only allowed a qualitative analysis (Figure S4). LA formation was observed only after addition of the last enzyme (LDH) but not in any of the preceding reactions. After addition of all enzymes, mostly mannose and smaller amounts of mannonate, glyceraldehyde, and KDG, including several unspecific products, were still visible. The initial yield of the proof-of-concept reaction was 18.9 ± 6.3% of the theoretical maximum with 3.78 ± 1.26 mM LA produced from 20 mM mannose.

### Validation and Optimization of One-Pot Reactions

Next, we performed one-pot reactions under varying parameters to identify the most important factors influencing the efficiency of the process. In order to investigate the effect of enzyme composition, the loading of each individual enzyme was reduced to 10% while keeping the other enzymes of the pathway at a constant loading (Table 2). Lower loads of AldT and LDH resulted in comparably low LA yields of 45.6 ± 3.6 and 45.5 ± 1.1% of the theoretical maximum, respectively. The most drastic effect was obtained when the ManD loading was reduced, which resulted in a LA yield of only 20.2 ± 0.7% of the theoretical maximum. This suggests that the ManD loading appeared to be the most critical in the one-pot reaction. No difference was observed between lower KDGA loading and the standard loading, indicating that a 10-fold reduction in KDGA could be used without compromising the final LA yield. The standard enzyme loading of 0.2 U/ml AldT, 0.1 U/ml ManD, 0.4 U/ml KDGA, and 11.6 U/ml LDH resulted in a LA yield of 71.5 ± 4.1% of the theoretical maximum from 5 mM mannose and NAD^+^. This enzyme composition was used for following one-pot reactions.

**Table 2:** Variation of each individual enzyme loading and its effect on LA yield of the theoretical maximum.

**Enzyme**	**Enzyme concentration [U/ml]**				**Standard** **enzyme loading**
AldT	0.02	0.2	0.2	0.2	0.2
ManD	0.1	0.01	0.1	0.1	0.1
KDGA	0.4	0.4	0.04	0.4	0.4
LDH	11.6	11.6	11.6	1.6	11.6
Yield (%)	45.6 ± 3.6	20.2 ± 0.7	68.6 ± 0.04	45.5 ± 1.1	71.5 ± 4.1
Titre (mM LA)	2.3 ± 0.18	1 ± 0.03	3.4 ± 0.002	2.3 ± 0.05	3.6 ± 0.21

*Red cells indicate decreased enzyme loading (10% of standard enzyme loading). Reactions contained 5 mM mannose and NAD^+^, 0.1 mM CoSO_4_ in 0.1 M NaP buffer at pH 7.0*.

Besides enzyme concentration, process duration and operational temperature directly influence enzymatic stability. An efficient process aims not only at a high yield but also high titres after a process-specific period of time. Half-life times for enzymes found in the literature are often acquired at higher temperatures but at assay times much shorter than those used in their actual operational conditions. In addition, thermostability depends very much on the concentration of the particular enzyme during the thermostability assay, which in many cases differ from the actual concentration used in the process. Therefore, in order to assess the operational stability of each enzyme in the process, enzymes were assayed at their standard enzyme loading, in the same buffer and at 50°C over a 20 h period (Figure S5). Under these conditions LDH and KDGA remained fully active over the entire assay period. AldT displayed a half-life of 9.8 h while retaining 30% of its initial activity after 20 h. The half-life of ManD was only 1.4 h and no activity could be detected after 6 h.

### Substrate and Cofactor Concentration

The initial substrate concentrations of mannose and in particular the cofactor NAD^+^ play important roles in the economic viability of the reaction cascade, since substrates and cofactors, beside enzymes, are major cost drivers in a cell-free reaction process. *In vivo*, endogenous cofactors are regenerated continuously, e.g., through coupled side reactions. However, *in vitro*, cofactors are supplied externally into a cell-free biocatalytic system and may considerably affect its cost-effectiveness. The cell-free reaction cascade presented here consisted of purified enzymes and, therefore, lacked the endogenous cofactors required to start and drive the first reaction of the reaction cascade. Accordingly, NAD^+^ has to be supplied at the beginning of the process in sufficient amounts to catalyze the initial oxidation of mannose to mannonate until it is recycled again by LDH at the end of the process. However, since the reaction cascade was assembled with thermostable enzymes and designed to operate at 50°C, cofactor thermal stability becomes an important factor to consider. Incubation of NAD^+^ at 60°C in HEPES-NaOH pH 8.0 over a 6 h period has been shown to result in 50% decomposition of the cofactor into ADP-ribose and nicotinamide (Honda et al., 2016). Krutsakorn et al. (2013) found that NAD^+^ and NADH levels decreased to about 50% during a reaction time of 4 h in 50 mM HEPES-NaOH pH 7.0 and supplemented additional cofactor to achieve maximum conversion. In another study, no degradation was observed in 50 mM HEPES-NaOH pH 7.0 at 60°C for 1 h (Ye et al., 2012). Besides its thermal instability, NAD^+^ has also been shown to inhibit certain enzymes such as LDH from *Thermus thermophilus* which was almost completely inactivated above a concentration of 0.5 mM (Ye et al., 2012).

**Figure 2:**
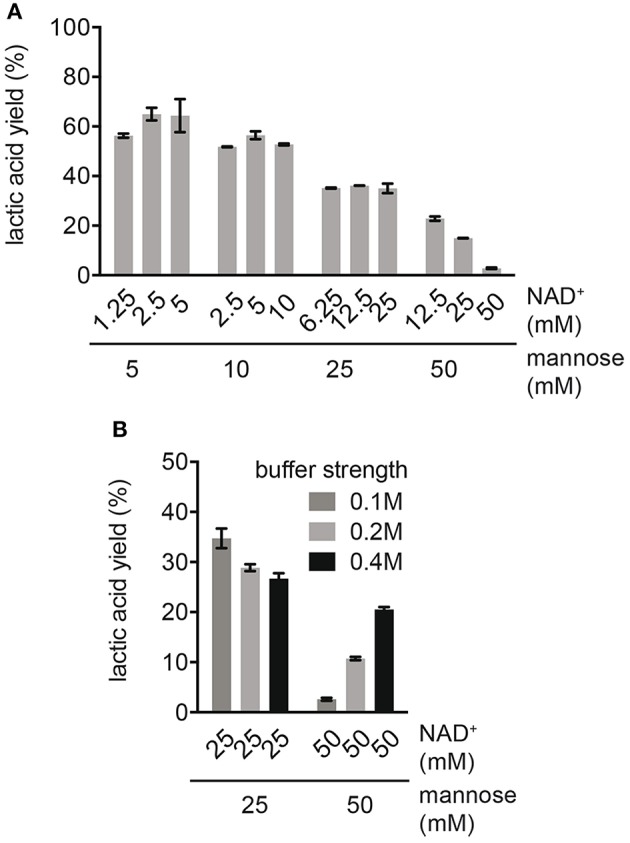
Effect of different mannose and cofactor concentrations on LA yield. **(A)** Reactions were performed in 0.1 M NaP buffer pH 7.0 with standard enzyme loading. **(B)** Effect of ionic strength on LA yield in reactions with high substrate concentrations. Reactions were performed with 25 and 50 mM mannose, equimolar NAD^+^ concentration and with standard enzyme loading.

In order to study the effects of cofactors on process performance, the production of LA was compared at different mannose concentrations in combination with varying NAD^+^ concentrations (Figure 2A). For reactions with varying NADH concentrations (e.g., 1.25, 2.5, and 5 mM NAD^+^) at an equal mannose concentration (e.g., 5 mM mannose), no major difference in LA yield was observed. This suggested that cofactor decomposition was not affecting LA yields at this stage. The same was true for reactions with higher mannose concentrations (10 and 25 mM mannose), indicating that NAD^+^ was efficiently recycled to NADH. However, independent of the cofactor concentration, an increase in mannose concentration caused a decrease in LA yield (Figure 2A). We investigated whether the decrease in yield at high substrate concentrations was caused by insufficient buffer capacity, since a high production of organic acids from sugar acidifies the reaction. The ionic strength of the buffer was increased from 100 to 400 mM in reactions with high substrate concentrations (25 and 50 mM mannose) at equal NAD^+^ concentrations (Figure 2B). In reactions with 50 mM mannose, enhancing the ionic strength increased the LA yield from 2.6 ± 0.3 to 20.5 ± 0.5% of the theoretical maximum, while the LA yield slightly decreased in reactions with 25 mM mannose. This suggested that only at very high mannose concentrations (50 mM) acidification of the solution was limiting the production of LA. For the decrease in LA yield in reactions with up to 25 mM mannose, there must been another reason. Although no substrate inhibition was observed in single enzyme kinetic studies (Figure S2), it is possible that intermediates other than the natural substrate of each enzyme act as inhibitor.

### LA Production From SCG

**Figure 3:**
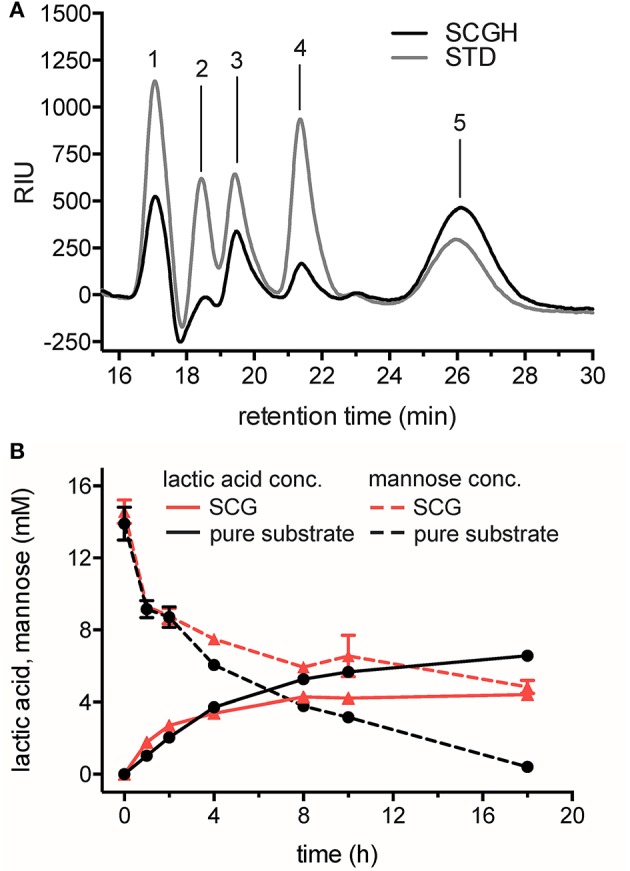
Hydrolysis of SCG and its conversion into LA using the cell-free reaction cascade. **(A)** Analysis of monosaccharides after dilute acid hydrolysis of SCG. STD, sugar reducing standards (gray line); SCGH, spent coffee grounds hydrolysate (black line). Retention times of standards are indicated. 1: D-glucose 2: D-xylose 3: D-galactose 4: L-arabinose 5: D-mannose. **(B)** Production of LA from mannose obtained from SCG (red lines) compared to production from pure mannose (black lines) in time-course experiments. Decrease of mannose (dashed lines) and increase of LA (solid lines) were monitored by HPLC-RID and -DAD analysis and quantified by mannose and L-lactic acid standards. Reactions from SCG hydrolysate and from pure mannose as a substrate contained 14.57 ± 0.7 mM and 13.9 ± 0.9 mM mannose, respectively, 3.75 mM NAD^+^, 0.1 mM CoSO_4_, and standard enzyme loading in 0.1 M NaP buffer pH 7.0. Reactions were performed in triplicate at 50°C. Error bars represent standard deviation of the mean.

The feasibility of the cell-free reaction cascade for the conversion of biomass to LA was tested with sugars obtained from SCG. In order to substitute purposely-refined sugar as substrate, we obtained mannose directly from the galactomannan present in SCG. SCG was dried, subjected to a standard dilute acid hydrolysis procedure and analyzed regarding its mannose content by HPLC (Figure 3A) (Sluiter et al., 2008). The mannose content of 100 mg SCG was found to be 28.7 ± 1.5 mg and is comparable to previous obtained values (Simões et al., 2009; Mussatto et al., 2011). However, due to harsh hydrolysis conditions only 24 ± 1.26 mg/100 mg SCG remained available for subsequent conversion. The hydrolysate was neutralized, concentrated and used as substrate for the cell-free enzymatic production of LA. The conversion with mannose obtained from SCG hydrolysis was compared to reactions with pure mannose by monitoring the decrease in mannose and the accumulation of LA over time (Figure 3B).

HPLC analysis of the reactions demonstrated that LA was produced successfully from SCG hydrolysate. In reactions with mannose obtained from SCG, 4.4 ± 0.1 mM LA was produced from 14.57 ± 0.7 mM mannose (30.3 ± 1.5% yield of the theoretical maximum), whereas in reactions with pure mannose as substrate, 6.6 ± 0.1 mM LA could be produced from 13.9 ± 0.9 mM mannose (47.4 ± 3.9% yield of the theoretical maximum). Based on the concentration of mannose remaining compared to the amount of product formed, it was evident that a significant amount of mannose was not converted to LA in reactions from SCG. In contrast, mannose was almost completely oxidized in reactions with pure mannose. Further, metabolite analysis of the SCG hydrolysate and single enzyme assays in presence of hydrolyzed SCG could help to identify potential enzyme inhibitors in the mannose-rich waste resource.

### Real-Time Tracking of the Cell-Free Reaction Cascade Using ^13^C NMR

**Figure 4:**
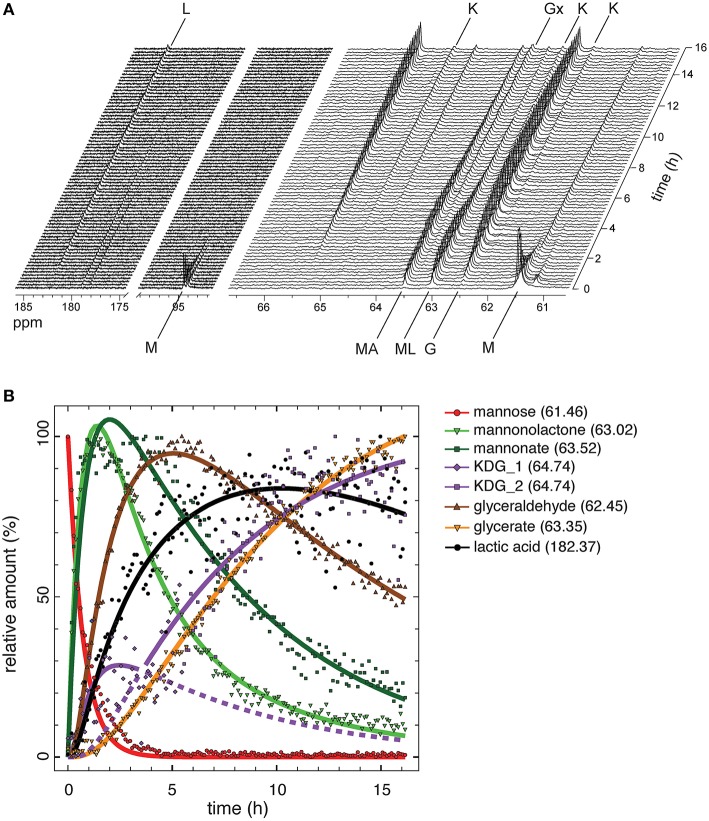
Flux analysis of the reaction cascade using NMR. **(A)** Real-time ^13^C NMR analysis of one-pot reaction performed with 10 mM D-[1,6-^13^C_2_]mannose and 10 mM NAD^+^ for 16 h. M, mannose; MA, mannonate; ML, mannonolactone; K, KDG; G, glyceraldehyde; Gx, glycerate; L, L-lactic acid. **(B)** Relative amounts of the intermediates derived from integration of areas for characteristic chemical shifts for each intermediate. Only integrals for the lower chemical shift of C6 compounds were analyzed. Integrals were fitted using DynaFit 4.0 (Kuzmič, 1996).

Monitoring of intermediate metabolites in cell-free reaction cascades is a crucial tool in understanding flux and identifying bottlenecks. Since the highest yield of the process was 71.5% of the theoretical maximum with 5 mM mannose and NAD^+^, the rest of the carbon was probably lost in incomplete or unspecific side reactions. Using several HPLC setups and conditions did not result in a clear separation of all intermediates (Figure S4). This has been shown previously to be a challenging task for similarly polar organic acids (Xie et al., 2018). To address this challenge, we performed reactions with ^13^C labeled substrate in combination with nuclear magnetic resonance (NMR) spectroscopy to trace every carbon during the reaction in the system. Reactions with D-[1,6-^13^C_2_]mannose were performed at 50°C over a time period of 16 h, while decoupled 1D ^13^C-NMR spectra were acquired every 6.5 min (Figure 4A). Chemical shifts appearing during the experiment, were allocated to intermediates by comparison to chemical shifts in 1D ^13^C-NMR spectra of unlabeled standards of each reaction intermediate (Table S2, Figure S7). Peak areas for each chemical shifts were integrated for all acquired spectra over the time of the reaction. The integrals for each chemical shift represent the relative amount of each intermediate in relation to the maximal integral occurring during the reaction (Figure 4B). Absolute intensity between peaks cannot be accurately used, as this would require longer acquisition times and a different pulse program to prevent ^1^H NOE enhancement of the ^13^C signal, which would also require longer acquisition times. However, the compromise of short acquisition times and use of relative maximum intensity still allows monitoring of the flux for each intermediate at a high time resolution.

D-[1,6-^13^C_2_]mannose was identified in ^13^C NMR by the doublet at 94.09/94.46 ppm representing the α- and β-anomers of D-mannose and a single peak at 61.46 ppm representing carbon 6 (Figure 4A). Both peaks decreased in their area very rapidly and mannose was fully consumed after 3 h (Figure 4B). The reaction products of the initial oxidation of mannose, mannonate and D-mannono-1,4-lactone, were identified by a chemical shift difference at their carbon 6. Both oxidation products appeared almost simultaneously, but the level of D-mannono-1,4-lactone decreased earlier in comparison to the free sugar acid, D-mannonate. This can be explained by the hydrolysis of the lactone into D-mannonate. About 20–25% of mannonate was still not converted after 16 h of incubation, which contributed to the loss of carbon in the cell-free conversion to LA. The next intermediate in the reaction, KDG, was represented by three chemical shifts, which were observed in the standard and in the one-pot reaction (Table S2). The most pronounced shift occurred at 64.74 ppm, while two smaller peaks were visible at 62.24 and 62.78 ppm. All integrals of chemical shifts for KDG demonstrate a biphasic accumulation of the compound. After a sharp increase and decrease during the first 5 h, KDG levels continued to accumulate linearly throughout the rest of the reaction. Close fitting of remaining reaction cascade intermediates was only possible by describing the integrals for of KDG with two separate mechanisms (Table S3, Figure 4B purple lines). A possible reason for the linear accumulation of KDG after the initial spike might be related to the low positive change in Gibbs energy (+6.2 kJ/mol) of the aldolase cleavage reaction. Although LDH is highly active and converts pyruvate rapidly, it has a lower affinity to pyruvate than KDGA (Table 1). Therefore, an aldol addition reaction of glyceraldehyde and pyruvate yielding KDG is likely to occur. Chemical shifts for the next intermediate, pyruvate, did not appear in the time-course reaction, since LDH was expected to convert its substrate rapidly into LA and also KDGA potentially converting pyruvate in a reverse reaction. The integral of the chemical shift at 62.45 ppm, representing glyceraldehyde, reached a maximum after ~6 h reaction time and then continued to decrease until the end of the reaction. Interestingly, we detected oxidation of glyceraldehyde presumably to glycerate (63.35 ppm), which was also found to appear spontaneously in the standard. Oxidation of glyceraldehyde could be caused by either by spontaneous oxidation or more likely by the aldohexose dehydrogenase. The final product LA accumulated right from the start of the reaction. The strongest increase in product levels was observed already in the first 1–2 h of the reaction, eventually reaching a steady state after approximately 9 h, which is in line with the observations made from HPLC analysis (Figure 3B).

The Maillard reaction has been described as a non-enzymatic browning reaction between amino- and carbonyl groups under heat (Maillard, 1912) that can affect the productivity of enzymatic pathways (Cheng et al., 2018). In our reaction cascade, mannose was incubated with proteins and phosphate-based buffers which are known to promote the Maillard reaction. Based on this, one would expect that that mannose degraded and enzymes were inactivated over the time of the reaction (Bell, 1997). However, we were able to exclude this effect as no rise in unspecific chemical shifts could be observed in a control reaction with mannose and bovine serum albumin incubated under the standard conditions and temperature used with the cell-free reaction cascade.

The process presented here does not fully exploit the carbohydrate substrate, since only pyruvate is converted to LA and glyceraldehyde remains unused. Guterl et al. (2012) demonstrated a recycling process for glyceraldehyde by implementation of an engineered aldehyde dehydrogenase and a promiscuous dihydroxy-acid dehydratase (Steffler et al., 2013; Carsten et al., 2015). The natural promiscuity of the enzyme allowed to convert 100% carbon toward KDG while keeping the NAD^+^/NADH ratio balanced. A similar recycling process could potentially be combined with the pathway presented in this work to enable a conversion of the complete carbon in mannose and avoid the accumulation and oxidation of glyceraldehyde.

The performance of the cascade can potentially be improved by implementing enzymes in a continuous flow setup using column reactors and tethered cofactors (Hartley et al., 2019). A continuous flow setup would circumvent inhibition of enzymes by cascade intermediates and help to alleviate substrate inhibition by high mannose concentrations. One way to overcome high production cost of enzymes and poor enzyme stability is to immobilize enzymes onto inorganic matrices, such as zeolite, using solid binding peptides (SBPs) (Care et al., 2017). While immobilization using a SBP onto zeolite was successful for AldT and LDH, expression of ManD and KDGA with SBP resulted in insoluble enzymes (data not shown). Using different types of immobilization for each enzyme might allow reuse of all enzymes and multiple runs of the reaction cascade.

## Conclusion

We successfully demonstrated a proof of concept for a synthetic reaction cascade to enzymatically convert mannose, a major constituent of SCG, into the platform chemical LA. To the best of our knowledge, the chosen route is the shortest enzymatic conversion of mannose into LA, so far. ^13^C NMR using labeled intermediates provided a useful tool to track the course of these multiple reactions and identify unexpected intermediates and side reaction products. The synthetic pathway is free of heat-labile phosphorylated compounds and requires only small amounts of NAD^+^ and Co^2+^. One major bottleneck of pathway operation and productivity is the low activity and thermostability of the enzyme, ManD, which might be improved using different enzyme engineering techniques. An advantage of the cell-free approach is the rapid assessment of pathway performance and identification of potential bottlenecks. Although the cell-free reaction cascade presented here is considered synthetic, its operation is in principle possible *in vivo* and resembles similar oxidative metabolisms such as the np-ED, the Weimberg or Dahms pathways (Weimberg, 1961; Budgen and Danson, 1986). By hetereologous expression of three genes (Ta0753, Ta0753, Ta1157) derived from *T. acidophilum* it could be shown that a metabolic pathway from mannose to pyruvate and glyceraldehyde is enzymatically feasible. As demonstrated previously the genes for the dehydrogenase and dehydratase are located adjacent to each other in the genome of *T. acidophilum* and other Thermoplasmatales (Kopp et al., 2019). Further transcriptional, proteomic or physiological data will help to test whether the hypothesized mannose pathway is active in *T. acidophilum* and other archaea. Beyond its potential physiological role in archaea, the cell-free reaction cascade presented here could also be implemented as an orthologous mannose metabolism in microbial hosts (e.g., *S. cerevisiae*), which might be beneficial in the bioproduction of different products from mannose-rich waste resources.

## Data Availability Statement

All datasets generated for this study are included in the article/Supplementary Material.

## Author Contributions

DK and AS: conceptualization and writing—original draft preparation. DK, RW, and AS: methodology, experimental design, writing—review, and editing. DK: data collection and data analysis. AS: supervision and project administration.

### Conflict of Interest

The authors declare that the research was conducted in the absence of any commercial or financial relationships that could be construed as a potential conflict of interest.
