# Supplementary material for: Cell-Free Enzymatic Conversion of Spent Coffee Grounds Into the Platform Chemical Lactic Acid
Source: Front Bioeng Biotechnol. 2019 Dec 3;7:389. doi: 10.3389/fbioe.2019.00389 (PMC6901390; doi:10.3389/fbioe.2019.00389)
Supplement: Supplementary file 1 [file Data_Sheet_1.pdf]

Supplementary Material

Cell-free enzymatic conversion of spent coffee grounds into the platform chemical lactic acid

Dominik Kopp[1], Robert D. Willows[2], Anwar Sunna[1,2] *

[1]Department of Molecular Sciences, Macquarie University, Sydney, New South Wales, Australia

[2] Biomolecular Discovery and Design Research Centre, Macquarie University, Sydney, New South Wales, Australia

*** Correspondence:**
A/Prof. Anwar Sunna
anwar.sunna@mq.edu.au

Table S1 Oligonucleotides used in the study. All oligonucleotides were synthesised by IDT DNA Technologies (Singapore). For oligonucleotide sequences, restriction sites are written in lowercase.

Oligonucleotide name	Gene amplified	Oligonucleotide sequence (5' –> 3')
AldT-fw	aldohexose dehydrogenase	TGATggatccCATGTTCAGCGATCTAAGGGA
AldT-rev	aldohexose dehydrogenase	ATTATgaattcCTCATTCTGGCGTGCTTATGG
Ta1157_EcoRI_fw	KDG aldolase	ATATTgaattcCTACAAGGGTATCGTAACGCC
Ta1157_HindIII_rev	KDG aldolase	TGATaagcttATTGCTTCTTCGGGATTTTG
BsLDH_BamHI_fw	L-lactate dehydrogenase	ATTATggatccTAAAAACAACGGTGGAGCCC
BsLDH_HindIIII_rev	L-lactate dehydrogenase	TGATATaagcttTCATCGCGTAAAAGCACGG

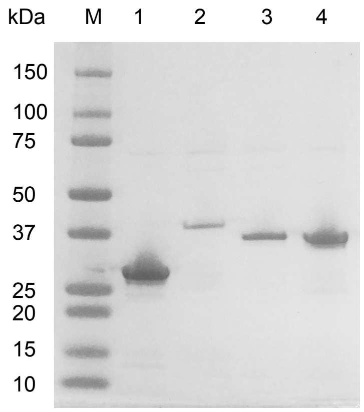

Figure S1 SDS-PAGE of the purified enzymes for the cell-free conversion of mannose to LA. AldT (1) and LDH (4) were expressed in *E. coli* BL21 cells. ManD (2) and KDGA (3) were expressed in BL21-CodonPlus (DE3)-RIL cells. Molecular weights of AldT, ManD, KDGA and LDH were 29 kDa, 42 kDa, 37 kDa and 36 kDa respectively. All enzymes were purified via their N-terminal His-tag using metal-affinity chromatography. M: Bio-Rad Precision Plus Protein Standard.

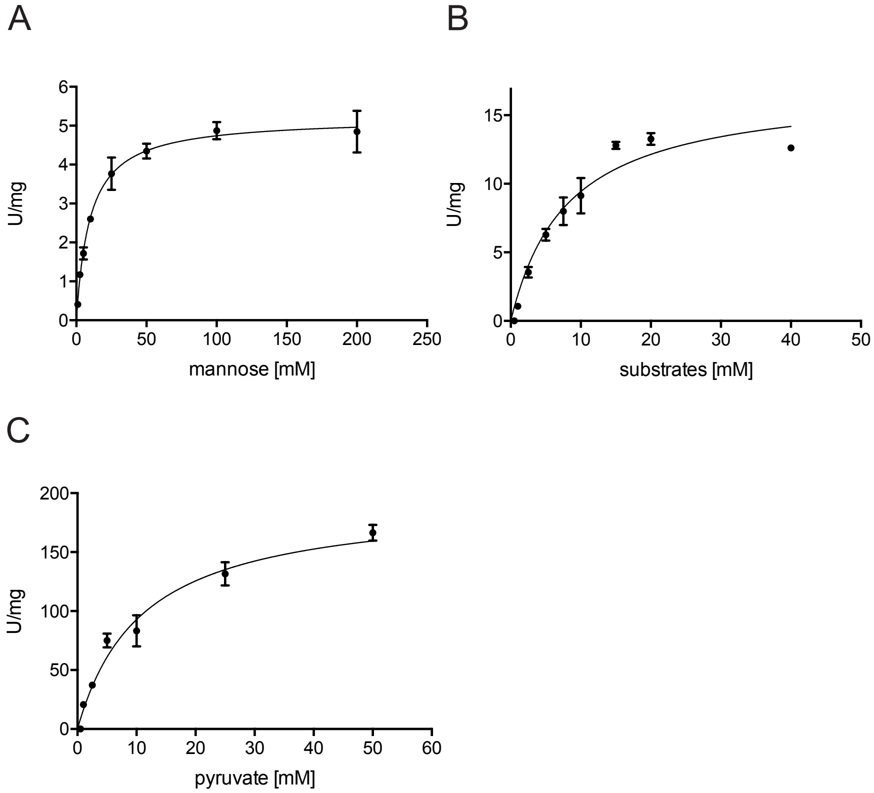

Figure S2 Kinetic data of purified enzymes. All data was fitted to Michaelis-Menten kinetics for one substrate. **A)** AldT for the substrate D-mannose **B)** KDGA measured in the direction of aldol addition with pyruvate and glyceraldehyde as substrates **C)** LDH for the substrate pyruvate. Curve fitting was performed with Prism 6 (GraphPad software). All experiments were performed in triplicate. Error bars represent standard deviation of the mean.

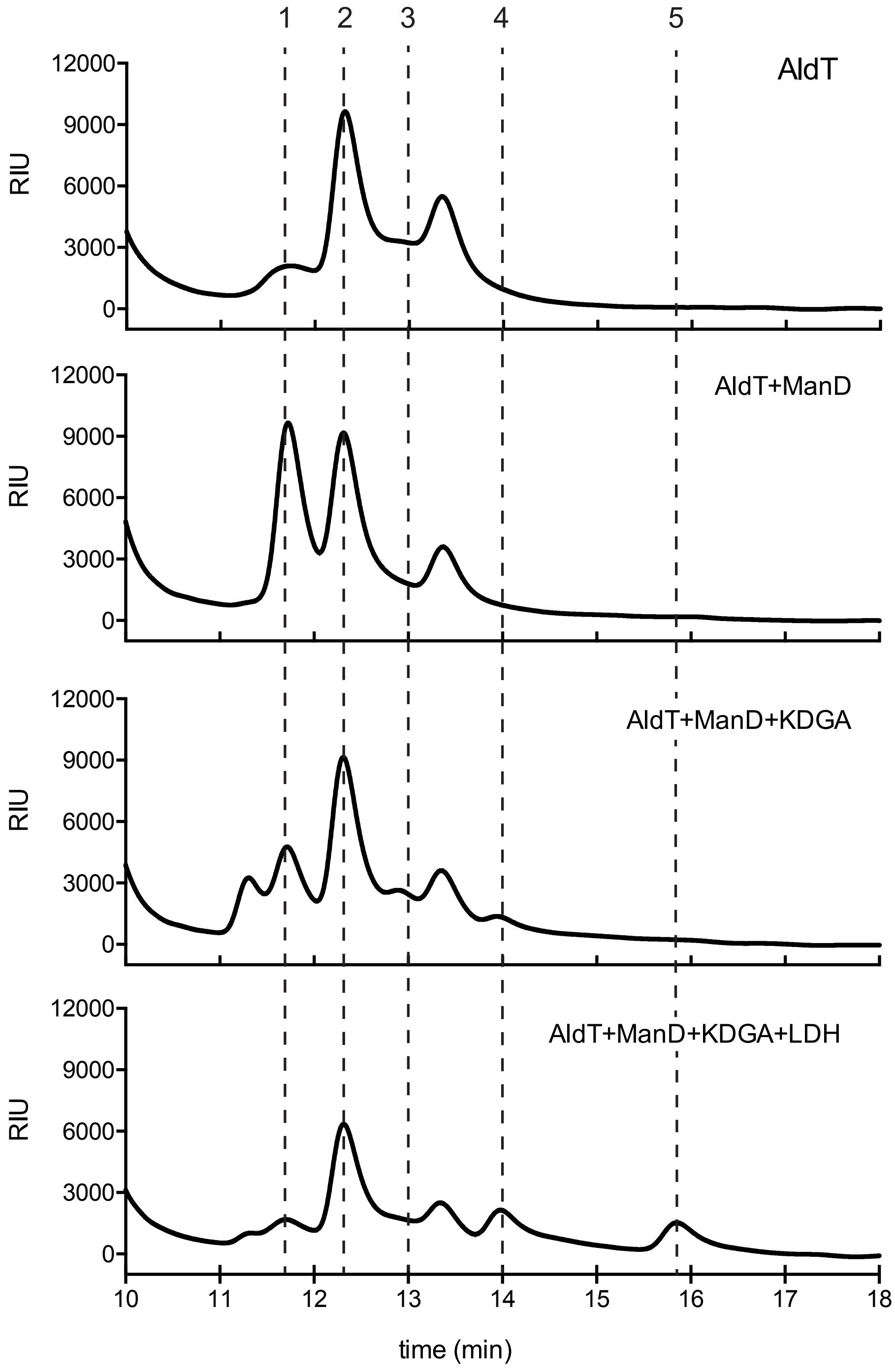

Figure S3 Exemplary sequential enzymatic pathway reactions demonstrating the activity of each enzyme and product formation after addition of all enzymes. Reactions contained a final concentration of 20 mM mannose and NAD^+ in NaP buffer pH 7.0 (50mM) and 0.1 mM $CoSO_4$. Enzyme loadings were: AldT: 0.16 U/ml, ManD: 0.07 U/ml, KDGA 0.51 U/ml, 5.8 U/ml LDH. Net retention times of standards for intermediates 1: KDG (11.69 min), 2: mannose (12.29 min), 3: pyruvate (13 min), 4: glyceraldehyde (14 min), 5: L-lactic acid (15.9 min). RIU: refractive index units. For separation of standards see Figure S4.

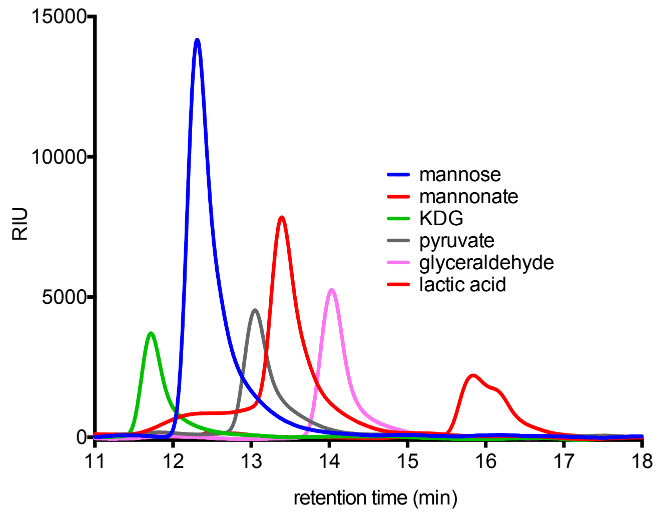

Figure S4 Separation of intermediates on organic acid column. 10 µl of 10 mM standards except from KDG (2.5 mM) were subjected to a Hiplex H^+ column connected to a RID (see Material and Methods for further details). Net retention times of standards are mentioned in Figure S3.

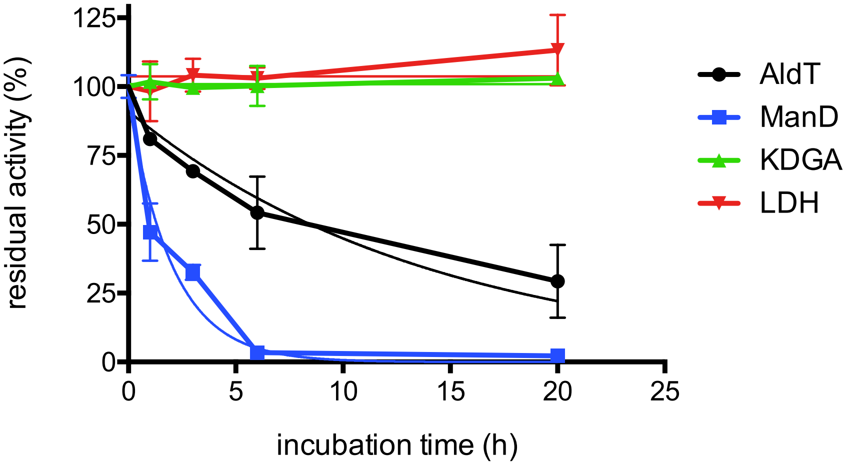

Figure S5 Thermostability of AldT (black line), ManD (blue line), KDGA (green line) and LDH (red line) at their standard enzyme loading at 50˚C over the duration of the process time. Residual activity is expressed relative to activity without any incubation at 50˚C.

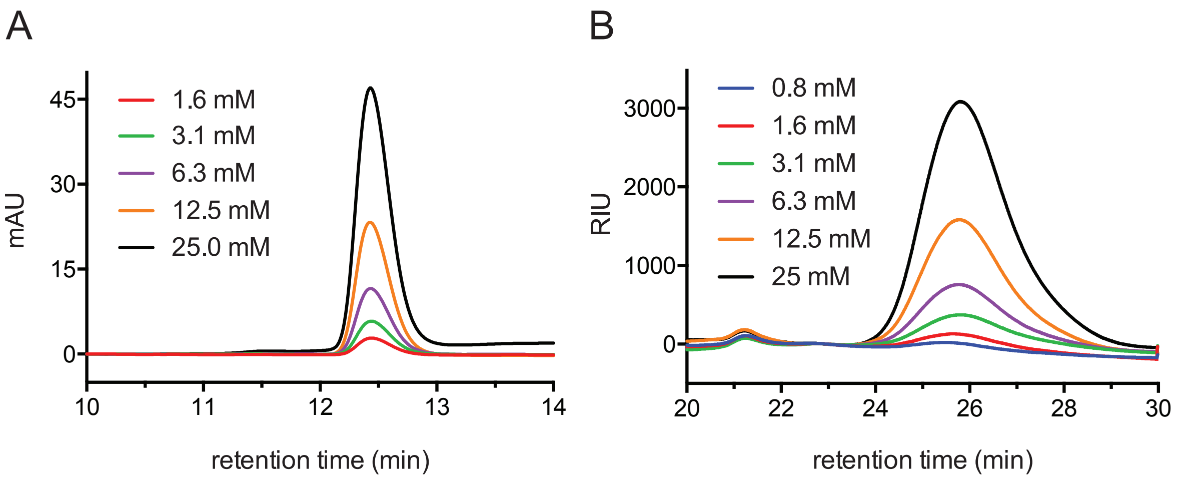

Figure S6 Standards used for the quantification of L-lactic acid **(A)** and mannose **(B)** in time course experiments for the conversion of mannose obtained from SCG and pure mannose into LA. Standards for LA were analysed on a Bio-Rad Aminex HPX-87H column and standards for D-mannose on an Agilent HiPlex Pb column (see Material and Methods for further details). Net retention times for L-lactic acid and D-mannose standards were 12.5 min and 25.8 min, respectively.

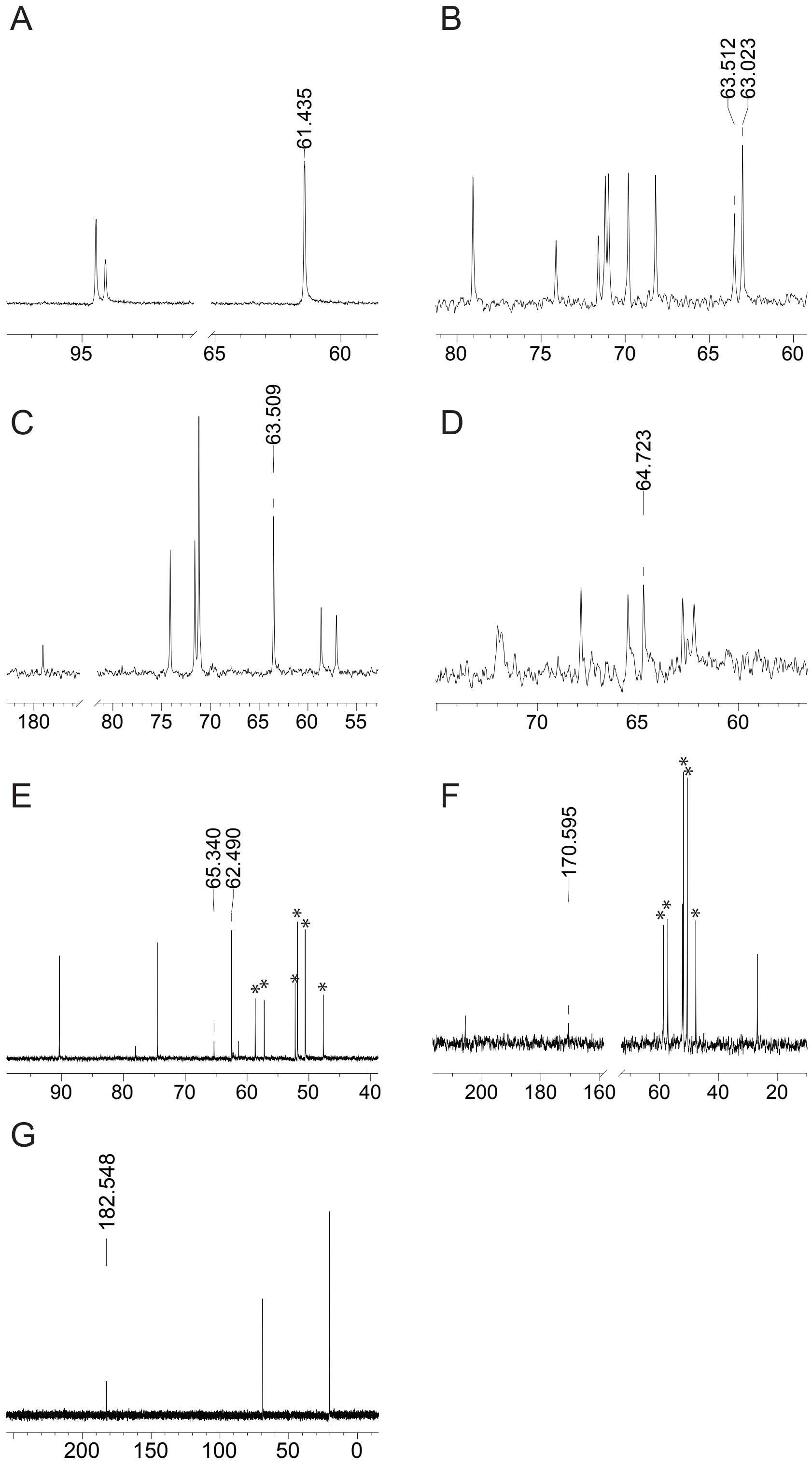

Figure S7 Decoupled 1D ^{13}C NMR spectra of standards. All values are expressed in ppm. All standards were acquired in 0.1 M NaP buffer pH 7.5 at 55°C. A: D-[1,6-$^{13}C_2$]mannose, B: mannono-1,4-lactone, C: mannonate, D: KDG, E: glyceraldehyde, F: pyruvate, G: L-lactic acid. Peaks labelled with numbers were found in real-time analysis of the reaction cascade, except for peak at 170.595 ppm for pyruvate. Peaks labelled with an asterisk (*) are buffer contaminants in these samples.

Table S2 Comparison of predicted chemical shifts, acquired chemical shifts of unlabelled standards and chemical shifts appearing in pathway reaction with D-[1,6-$^{13}C_2$]mannose and 10 mM NAD^+. Standards were obtained in the same buffer as the reaction took place as described in Material and Methods. n.d.: not detected. Prediction was performed with ChemDraw Professional 16.0 software.

	prediction		pathway reaction		standards	
Carbon atom	C1	C6	C1	C6	C1	C6
mannose	98.5	62.2	94.463 (α)<br>94.087 (β)	61.459	94.463 (α)<br>94.087 (β)	61.435
mannonate	176.2	64.4	179.002	63.518	n.d.	63.509
mannonolactone	175.3	64.6	178.168	63.016	n.d.	63.024
KDG	63.8	162.8	62.24<br>62.784<br>64.736	-	62.228<br>62.782<br>64.723	n.d.
pyruvate	-	162.8	-	-	-	170.595
glyceraldehyde	59.2	-	62.447<br>65.345	-	62.490<br>65.340	-
L-lactic acid	-	183	182.367	-	-	182.548

Table S3 Reaction mechanisms used in the script for DynaFit. DynaFit performed nonlinear least-squares regressions to fit integrals of chemical shifts for each intermediate. Kinetic constants (k) are optimised by DynaFit; x: unknown compounds.

mannose ---> mannonate	k1
mannonate ---> KDG_1	k2
mannose ---> mannonolactone	k3
mannonolactone <==> mannonate	k4, k5
KDG_1 --> pyruvate + glyceraldehyde	k6
pyruvate + glyceraldehyde --> KDG_2	k7
glyceraldehyde ---> glyceraldehyde_oxidised	k8
glyceraldehyde_oxidised --> x	k9
pyruvate ---> lactic_acid	k10
lactic_acid ---> x	k11
